# Nucleotide and Amino Acid Analyses of Unique Infectious Bronchitis Virus (IBV) Variants from Canadian Poultry Flocks with Drop in Egg Production

**DOI:** 10.3390/genes15111480

**Published:** 2024-11-17

**Authors:** Muhammad Farooq, Ahmed Ali, Mohamed S. H. Hassan, Mohamed Faizal Abdul-Careem

**Affiliations:** 1 Faculty of Veterinary Medicine, University of Calgary, Health Research Innovation Center 2C53, 3330 Hospital Drive, NW, Calgary, AB T2N 4N1, Canada; muhammad.farooq3@ucalgary.ca (M.F.); ahmed.ali@ucalgary.ca (A.A.); 2 Department of Pathology, Faculty of Veterinary Medicine, Beni-Suef University, Beni Suef 62511, Egypt; 3 Department of Avian and Rabbit Medicine, Faculty of Veterinary Medicine, Assiut University, Assiut 71515, Egypt; msh.hassan@ucalgary.ca

**Keywords:** infectious bronchitis virus, recombination, unique variants, S1 glycoprotein, N-glycosylation

## Abstract

Background/Objectives: Infectious bronchitis (IB) is a highly infectious avian disease caused by the infectious bronchitis virus (IBV). The disease causes lesions mainly in the respiratory, reproductive, and renal systems and has a significant economic impact on the poultry industry worldwide. Methods: We discovered two unique IBV isolates (T-62: PP737794.1 and CL-61: PP783617.1) circulating in Canada and molecularly characterized them. Results: The phylogenetic analysis revealed that the IBV isolates belong to genotype I and fall between lineages 25 and 7. Further analysis of the T-62 IBV isolate indicated that it is a potential recombinant of the Iowa state isolate (IA1162/2020-MW) and that the CL-61 strain of the IBV is also a recombinant IBV with the Connecticut (Conn) vaccine strain as its major parent. The S1 glycoprotein of the CL-61 and T-62 strains of the IBV had 85.7% and 73.2% amino acid (aa) identities respectively compared to the Conn vaccine strain. There were 67 and 129 aa substitutions among the S1 glycoprotein of the CL-61 and T-62 strains of the IBV compared to the Conn vaccine, respectively. Importantly, two and nineteen of these aa variations were in hypervariable regions 1 (HVR1) and HVR3. Finally, the two IBV isolates possessed a higher affinity for the sialic acid ligand compared to the DMV/1639 and Mass/SES IBV strains. Conclusions: Genetic recombination in the IBV results in the continual emergence of new variants, posing challenges for the poultry industry. As indicated by our analyses, live attenuated vaccine strains play a role in the genetic recombination of the IBV, resulting in the emergence of variants.

## 1. Introduction

Infectious bronchitis (IB) is an economically significant viral disease of poultry worldwide. IB is caused by the infectious bronchitis virus (IBV) and is characterized by acute respiratory distress, decreased egg production, increased mortality, and poor weight gain in infected birds [1]. Various strains of IBV have been identified and isolated from IB outbreaks in Canada. In the eastern region of the country, Delmarva (DMV)/1639 is the dominant prevalent strain, while the Massachusetts (Mass)-type strain has been isolated from the outbreaks in Western Canada [2,3]. Based on the history of IB outbreaks, the IBV variants present in Canada can be categorized into five groups: vaccine-like viruses; Canadian variants such as Qu_mv; US variants such as DMV/1639/11, California/1734/04, and CU/82792/GA98; Eurasian variants such as 4/91; and “unique variants” that do not show close similarity to any previously described IBV strains [4]. 

Mutation and recombination events during viral replication have been identified as key factors underlying the generation of genetic diversity in the IBV [5]. The IBV has a high potential to change either through spontaneous mutations, where only a few amino acid (aa) changes in the spike (S) protein can generate new variants, or through recombination resulting in major changes in the genome affecting both structural and nonstructural (Nsp) proteins [6]. Unique recombinant IBV variants have been isolated from backyard poultry in Kenya [7]. Similarly, according to recently published data from China, recombinant IBV strains have emerged and are prevalent, and this has been shown by analysing data from 13 outbreaks [8]. Recombination has been a major force in the emergence of multiple recombinant strains in Australia for at least 20 years [9]. Furthermore, in Canada, evidence of recombination in the *S1 gene* of DMV/1639 from the Connecticut (Conn) vaccine strain was noted [2]. Subsequently, new genotypes, serotypes, and prototypes have emerged that can evade host immunity and vaccination efforts, further complicating IB control measures [10,11]. 

In 2016, a criterion for the classification of the IBV was proposed, which is based on variations in the nucleotide sequence of the *S1 gene*. A genotype classification is based on 30% and 31% uncorrected pairwise variations in the S1 nucleotide and aa sequences, respectively. A strain falls into a different lineage when the S1 nucleotide and aa variation is at least 13% and 14%, respectively. As of May 2024, sequencing and phylogenetic analyses have resulted in the classification of the IBV into eight major genotypes, comprising 37 viral lineages and several inter-lineage recombinants [12,13,14,15]. 

The structural glycoprotein (S) is one of the key proteins on the surface of the virus. The S protein is known to be post-translationally cleaved by the host proteases into S1 and S2 subunits at the furin-specific site, which is between the RRFRR and HRRR aa. The cleavage of the S protein increases the binding affinity of the virus to the α 2,3-linked sialic acid residues of the host [16]. The S1 subunit binds to the receptor with the help of the receptor-binding domain (RBD) that specifically recognizes and attaches to the host cell receptors, whereas the S2 subunit facilitates the fusion of the viral envelope with the host cell membrane [1]. Three hypervariable regions (HVRs) are found within the S1 subunit: HVR 1 from aa positions 38 to 67, HVR 2 from aa 91 to 141, and HVR 3 extending from aa 274 to 387 [17,18]. It has been observed that high mutation rates and recombination events take place within the S1 region of the S gene [19,20,21,22]. 

The process of N-glycosylation in viral proteins is key factor in defining virulence and tissue tropism [23]. In coronaviruses, the glycosylation sites within the M and S proteins could influence viral fusion, receptor affinity, and antigenicity [24,25,26,27]. Variations in the N-glycosylation sites of the viral protein can impact the receptor binding affinity of the virus, which can result in variations in the host immune response regarding virus detection and clearance [28,29,30]. The N-glycosylation sites of the receptor-binding domain (RBD) of SARS-CoV result in reduced binding to the host defense peptide mannose-binding lectin, potentially affecting the immune reaction to SARS infection [8]. In another study, the N-glycosylation of the influenza virus affected T cell activation and cytokine production [31]. The envelope proteins of the envelope viruses, such as human immune deficiency virus (HIV), influenza virus, IBV, severe acute respiratory syndrome (SARS), and SARS-CoV, are deeply glycosylated. The presence of high glycans can mask the viral antigenic epitopes that may avoid recognition by neutralizing antibodies and lead to immune escape [32]. 

Despite the application of regular vaccines, novel IBV variants continue to emerge leading to significant economic damage to the global poultry industry [33]. This study provides genomic analyses of the unique IBV variants that have emerged in Eastern Canadian poultry flocks. In this analysis, we predict various characteristics of the unique IBV variants, including their ability to cause potential outbreaks and their adaptability to host receptors.

## 2. Materials and Methods

### 2.1. Case History

The samples from IBV-infected flocks in Eastern Canada were provided by the Animal Health Laboratory of the University of Guelph, Canada, and stored at −80 °C until they were processed. The T-62 isolate was detected in cecal tonsil samples from a commercial layer flock with a history of sudden reductions in egg production, while the CL-61 isolate was detected in cloacal swab samples of a small poultry flock with a history of respiratory clinical manifestations.

### 2.2. RNA Extraction and cDNA Synthesis

Total ribonucleic acid (RNA) was extracted from the cecal tonsils and swab samples using the Trizol^®^ reagent (Invitrogen Canada Inc., Burlington, ON, Canada) following the company’s recommendations. The concentration and quality of the extracted RNA were determined based on 260/280 nm wavelength absorbance using a Nanodrop1000 spectrophotometer (ThermoScientific, Wilmington, DE, USA). A total of 1000 µg of RNA (in 20 µL) from the sample was converted into cDNA using a High-Capacity cDNA Reverse Transcription Invitrogen Kit (Life Technologies, Carlsbad, CA, USA) following the company’s protocol.

### 2.3. Real-Time PCR

The SYBR green based quantitative PCR (qPCR) assay was conducted using *nucleocapsid (N) gene* primers (Fw-5′GACGGAGGACCTGATGGTAA-3′ and Re-5′CCCTTCTTCTGCTGATCCTG-3′), following previously described methods [34]. An in-house IBV *N-gene* plasmid served as the standard for making a 10-fold serial dilution curve. The reaction was carried out with Fast SYBR^®^ Green Master Mix (Quntabio^®^, Beverly, MA, USA) in a 20 µL total volume, including 10 µL of master mix, 100 ng of cDNA template in 2 µL and 0.5 µL each of forward and reverse primers (10 nM), and 7 µL of molecular-grade water. The qPCR was performed in 96-well plates with the following conditions: initial denaturation at 95 °C for 20 seconds (s), followed by 40 cycles at 95 °C for 3 s and 60 °C for 30 s for both annealing and extension, and a final extension at 60 °C for 10 min. The specificity was confirmed by a melting curve analysis conducted at 95 °C for 10 s with a 0.5 °C increment every 5 s to check for primer dimers.

### 2.4. Sequencing

The cDNA containing higher IBV genome copies (Ct value ≤ 20) was dispatched to the Veterinary Diagnostic Centre (VDC). Whole genome sequencing was performed by the Faculty of Veterinary Medicine, University of Montreal, Montreal, QC, Canada, utilizing an Illumina MiSeq platform (Illumina Corp, San Diego, CA, USA). The whole genomes of both unique IBV sequences were submitted to the National Center for Biotechnology Information (NCBI) GeneBank for general availability. The description of submission is (IBV/Ck/Can/18-T-62; GeneBank: PP737794.1) and (IBV/CK/CAN/18-CL-61; GeneBank: PP783617.1). 

### 2.5. Preparation of Reference Sequences

Following quality control steps, the sequences of the IBV mapping were performed against the Beaudette reference strain (accession number NC_001451) using the BLAST platform from the NCBI browser, which was used for the identification of the percent similarities of the obtained sequences available at GenBank. Both the whole genomes and *S1 genes* were blasted separately, and the highest similar sequences of the IBV from Canadian vaccines and those prevalent in North America were retrieved [35,36,37]. In addition, representative sequences from each genotype and lineage, as classified by Valastro et.al., were also included in the list [14].

### 2.6. Alignments and Phylogenetic Tree

Using Geneious^®^ software, version 10.2.6 (https://www.geneious.com/ accessed on 6 May 2024), a pairwise alignment of the *S1 gene* sequence was carried out with the fast Fourier transformation (MAFFT) algorithm [38]. The multiple sequence alignment, construction of the phylogenetic tree, and similarity distances were plotted with Geneious^®^ V 10.2.6 (alignment file attached in SS1, SS2). The phylogenetic tree was generated using the maximum likelihood method with 1000 bootstrap replicates in the MEGA Version 10 software. The visualization of the edited tree was performed with the iTOL v5 program.

An online tool ORF-finder (https://www.ncbi.nlm.nih.gov/orffinder/ accessed on 4 June 2024) was used to identity the open reading frames in the sequences and to compare them with the reference Baudette strain. The whole genome sequences were divided into genes based on the reference gene and ORF-finder’s results. The *genes 1a*, *1ab*, and *S* (*S1* and *S2*), along with *3a*, *3b*, *E*, *M*, *5a*, *5b*, and *N*, were blasted separately in the NCBI gene platform, and the first 2 highest similar sequences were imported to the list.

### 2.7. Detection of Recombination

The RDP β 5.53 (Recombination Detection Program) was used to identify the recombination events and potential breakpoints [39]. All 7 algorithms of the software with the full exploratory recombinant scan option were applied to the relevant whole genome and S1 gene sequences of IBV: 1, RDP (R); 2, GENECONV (G); 3, BootScan (B); 4, MaxChi (M); 5, Chimeara (C); 6, SiScan (S); 7, 3Seq (T) [40]. The T-62 and CL-61 sequences were kept as query sequences, and other strains, including vaccine strains, strains common in Canada and the US, and strains from the lineages closer to T-62, were used as reference strains in this analysis. Only those recombinant vents were considered significant that were inferred by at least seven different recombination searching algorithms on the default settings of the RDP5 software with a *p*-value of less than 5 × 10^−6^) [40,41].

### 2.8. S1 Glycoprotein Amino Acids Multiple Sequence Alignment

The homology analysis of the aa sequence of the S1 glycoprotein was obtainable using the fast Fourier transformation (MAFFT) [38], which was installed as a plugin in the Geneious^®^. The S1 glycoprotein of the two IBV isolates was aligned with the Mass H120 and Conn vaccinal strains, which were retrieved from the GenBank (https://www.ncbi.nlm.nih.gov/) under accession numbers FJ888351 and KF696629, respectively. Furthermore, the M41 strain was also obtained from GenBank under accession number AY851295, which contains the amino acids that are required to bind to the respiratory tract of chickens.

### 2.9. Analysis of Selection Pressure Sites in the S1 Glycoprotein Codon

The individual codons of the S1 glycoprotein were subjected to purifying (negative) or diversifying (positive) selections. Two approaches were used, fixed-effect likelihood (FEL) and fast unbiased Bayesian approximation (FUBAR), for this analysis [42]. To assess the selective pressure on the *S1 gene*, the ratio of nonsynonymous (dN) to synonymous (dS) substitution rates (dN/dS ratio) was estimated. The mixed effects model of evolution (MEME) was conducted to analyze the episodic diversifying selection [43]. The resides were considered to be under purifying or diversifying selection only if detected by the two used methods. The FEL and MEME methods had a significance level of *p* < 0.1, while the FUBAR method had a posterior probability threshold of 0.9. The Datamonkey online server (http://www.datamonkey.org) served as the platform for all analyses.

### 2.10. Construction and Validation of 3D Structures of the S1 Glycoprotein

The 3D models of the S1 glycoprotein of the current IBV isolates were generated using the I-TASSER server. This online platform is primarily based on the aa sequence, and it provides predictions of secondary, tertiary, and 3D structures, along with some uses relating to protein function [44]. Furthermore, the S1 glycoproteins of two circulating Canadian IBV strains, including DMV/1639 (IBV/Ck/Can/17-036989) and Mass/SES (15AB-01) [2,3], were also constructed; these sequences were recovered from the Genbank (accession numbers MN512435 and MH539771, respectively). The quality of the 3D constructs was validated by the Ramachandran plot that was created using the Vadar server [45]. By employing the UCSF Chimaera software [46], the similarity among the 3D modelled structures was accomplished.

### 2.11. Potential N-Glycosylation Site Analysis in the S1 Glycoprotein

The construed aa sequences of the T-62 and CL-61 IBV strains and Mass/SES were submitted to the NetNGlyc server 1.0 [47] to predict the N-glycosylation sites. The standard cutoff point of 0.5 was implemented to denote the putative N-glycosylation sites. The mapping of the N-glycosylated aa residues was performed using the PyMOL software (version 2.5.7).

### 2.12. Examination of S1 Glycoprotein–Sialic Acid Interactions by Molecular Docking

The sialic acid was used as a ligand, and it was retrieved in SDF format (PubChem CID 444885) from the PubChem database [48]. Afterwards, Avogadro software [49] was used to prepare the ligand by minimizing energy. The S1 glycoproteins in PDB format were prepared using the Swiss PDB viewer [50] to minimize their energy. After the preparation of the proteins and ligands, AutoDock Vina, which is incorporated into the CB-Dock server [51], was used blindly for the molecular docking. The docking results were viewed in the Discovery Studio software to show the bond and non-bond interactions.

## 3. Results

### 3.1. IBV Detection in Clinical Samples

The qPCR results showed that CL-61 and T-62 had a cycle threshold (Ct) value of 19.2 and 19.4, respectively, which facilitated the sequencing without propagation of the IBV isolates.

### 3.2. Alignment and Phylogenetic Tree

According to the classification criteria established by Valarstro (2016), an analysis of our sequences revealed these two isolates of the IBV represent unique variants. This classification is based on the observation of enough variations within the genome and aa sequences of the S1 structural protein. We observed more than 13% of variations in both isolates (14.24% in the T-62 and 13.08% in the CL-61 strain of the IBV) in the nucleotides and more than 14% (15.7% in the T-62 and 14.05% in the CL-61 strain of the IBV) in the aa, in comparison to all other available IBV sequences in the NCBI database. A list of the reference IBV sequences representing all the genotypes and lineages used as a reference for alignment is provided in Supplementary Table S1. 

**Figure 1:**
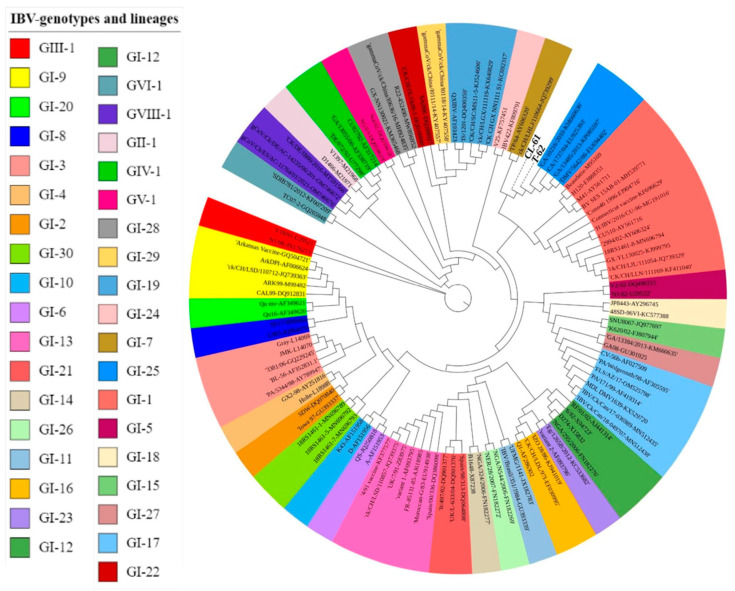
Phylogenetic tree including unique isolates T-62 and CL-61 (denoted without background color) and 114 reference strains of IBV *S1 gene* sequences. The design is constructed with the maximum likelihood method with 1000 bootstrap replicates in MEGA 11 software. The colors and design of the tree were performed with the iTol online tool.

Likewise, in the phylogenetic analysis (Figure 1), the two IBV isolates from our study were positioned in IBV genotype I classification. These isolates were positioned on the phylogenetic tree between lineage 25 and lineage 7. Furthermore, the two isolates from our study fall on two different branches showing the variation between the isolates within themselves. The T-62 strain of the IBV made a clade with the lineage 25 and the CL-61 strain of the IBV was independent (depicted as bold and colorless in Figure 1). 

The total nucleotide coverage for CL-61 and T-62 was 27,577 and 27,617, respectively. There was not much variation between the genes in the whole genome coding for the structural and Nsp proteins of the two unique isolates. Most of the genes bear an identical number of sequences in nucleotides and code for a similar number of aa, though the position of the genes varied between the two isolates (Table S1). The S1 and 6b genes of CL-61 had four and six aa less than the strain in comparison. CL-61 had a deletion of three aa in its critical site that binds to chicken respiratory epithelium, as shown in Supplementary Table S2.

### 3.3. Recombination Analysis of the Whole Genome of the T-62 Strain of the IBV

**Figure 2:**
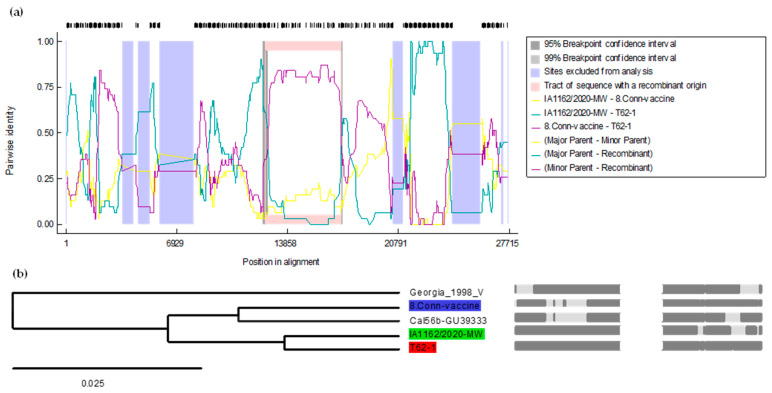
Recombination analysis of the T-62 IBV isolate based on the whole genome sequence using the RDP5 software. In (**a**), T-62 serves as the query sequence, while the lines show different pairs of analysis for recombination: yellow (Iowa state, Conn vaccine), green (Iowa state, T-62), and purple (Conn vaccine, T-62). In (**b**), the recombinant tree is presented, with the red area representing T-62 IBV as the recombinant, the green area indicating the Iowa state isolates (IA1162/2020-MW) as the major parent, and the blue area showing the Conn vaccine strain as the minor parent. Grey lines indicate regions of recombination. Dark grey areas represent consensus sequences. Light grey areas highlight breakpoints, indicating specific points where recombination has occurred. The Shimodaira–Hasegawa tree topology test yielded a *p*-value of <0.0001, based on 100,000 bootstrap replicates.

In the phylogenetic analysis, both unique variants of the IBV have a common ancestor but are located on different branches. A recombinant analysis for both the IBV isolates was performed separately. For the whole genome and *S1 gene*, the sequences of various IBV strains, including vaccine strains, strains common in Canada and the US, and strains from the lineages closer to the T-62, were used for the RDP 5 software. The whole genome sequence of the T-62 isolate of the IBV showed that T-62 is a potential recombinant strain with the major parent of its genome coming from the Iowa state isolate (IA1162/2020-MW) with a 97% similarity and the minor parent Conn vaccine strain with a 99.6% similarity, as shown in Figure 2a. The recombination fragment was noted in the 1ab gene from the whole genome analysis. The starting and ending breakpoints of the recombinant fragment were 12,490 and 17,228 with a 99% confidence interval in the alignment. The multiple comparison probabilities, both corrected and uncorrected, were 8.104 × 10^−5^ and 8.104 × 10^−4^, respectively. The results, as shown in Figure 2 and Supplementary Table S3, were confirmed with all seven statistical tools of RDP5 (R, G, B, M, C, S, and T).

### 3.4. Recombination in the S1 Gene of the T-62 Strain of the IBV

**Figure 3:**
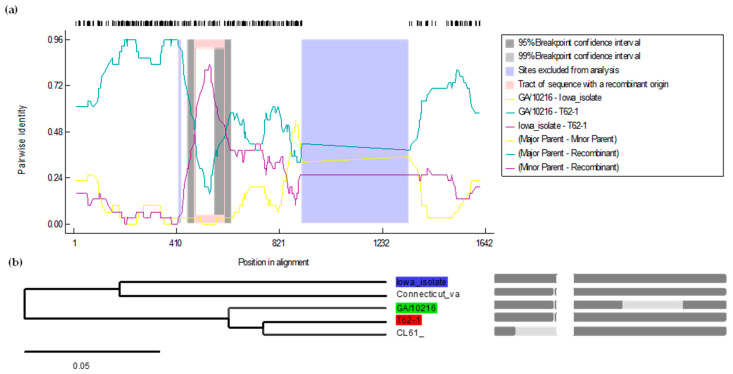
Recombinant analysis of the T-62 IBV isolate based on the *S1 gene* sequence using the RDP5 software. In (**a**), T-62 IBV serves as the query sequence, while the lines show different pairs of analysis for recombination: yellow (Georgia GA/10216, Iowa state), green (Georgia GA/10216, T-62), and purple (Iowa state, T-62). In (**b**), the recombinant tree is presented, with the red area representing T-62 IBV as the recombinant, the green area indicating the Iowa state isolates (IA1162/2020-MW) as the major parent, and the blue area showing the Conn vaccine strain as the minor parent. Grey lines indicate regions of recombination. Dark grey areas represent consensus sequences. Light grey areas highlight breakpoints, indicating specific points where recombination has occurred. The Shimodaira–Hasegawa tree topology test yielded a *p*-value of <0.0001, based on 100,000 bootstrap replicates.

Separate bioinformatics algorithms were used to create a consensus tree based on the *S1 gene* nucleotide sequences. Interestingly, in the whole genome, the recombination was observed in 1ab, whereas the analysis of the *S1 gene* revealed that a fragment in the *S1 gene* has been recombined. The major parent in the *S1 gene* of the T-62 isolate is the Georgia strain (GA/10216) and the minor parent is the Iowa state isolate (IA1162/2020-MW) with 90.4% and 96% nucleotide similarities, respectively. The multiple comparison probabilities, both corrected and uncorrected, were 7.135 × 10^−13^ and 7.135 × 10^−12^, respectively. The analysis based on the *S1 gene* showed nucleotide recombination at the starting and ending breakpoints in the *S1 gene* at nucleotide positions 478 and 604, respectively, with a 99% CI. The nucleotide region recombinant breakpoints did not contain any of the three hypervariable regions of the *S1 gene* (Figure 3).

### 3.5. Recombinant Analysis of the Whole Genome of the CL-61 Strain of the IBV

**Figure 4:**
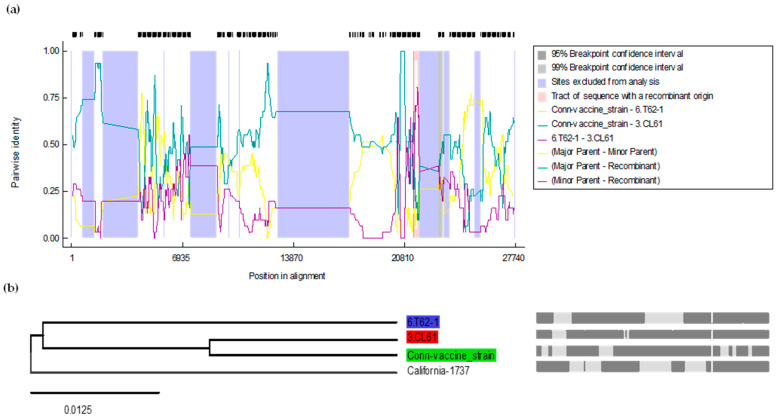
Recombinant analysis of the CL-61 isolate of the IBV based on the whole genome sequence using the RDP5 software. In (**a**), CL-61 serves as the query sequence, while the lines show different pairs of analysis for recombination: yellow (Conn vaccine, T-62), green (Conn vaccine, CL-61), and purple (T-62, CL-61). In (**b**), the recombinant tree is presented, with the red area representing CL-61 IBV as the recombinant, the green area indicating the Conn vaccine as the major parent, and the blue area showing T-62 as the minor parent. Grey lines indicate regions of recombination. Dark grey areas represent consensus sequences. Light grey areas highlight breakpoints, indicating specific points where recombination has occurred. The Shimodaira–Hasegawa tree topology test yielded a *p*-value of <0.0001, based on 100,000 bootstrap replicates. Recombination in *S1 gene* CL-61 strain of IBV.

The whole genome sequences of various IBV strains, including vaccine strains, strains common in Canada and the US, and strains from the lineages closer to the CL-61 strain of IBV, were used in the RDP5 software. The whole genome sequence of the CL-61 isolate showed that the CL-61 strain of IBV is a potential recombinant strain with the major parent of its genome coming from the Conn vaccine strain (96.4% similarity) and the minor parent T-62 strain of the IBV (90.4% similarity) isolate used in this study, as shown in Figure 3a. The recombination fragment was noted in the *S1 gene* from the whole genome analysis. The starting and ending breakpoints of the recombinant fragment were 21,416 and 21,750 with a 99% CI in the alignment. The multiple comparison probabilities, both corrected and uncorrected, were 8.672 × 10^−17^ and 8.672 × 10^−16^, respectively. The results, as shown in Figure 4 and Supplementary Table S4, were confirmed with all seven statistical tools of RDP5 (R, G, B, M, C, S, and T).

**Figure 5:**
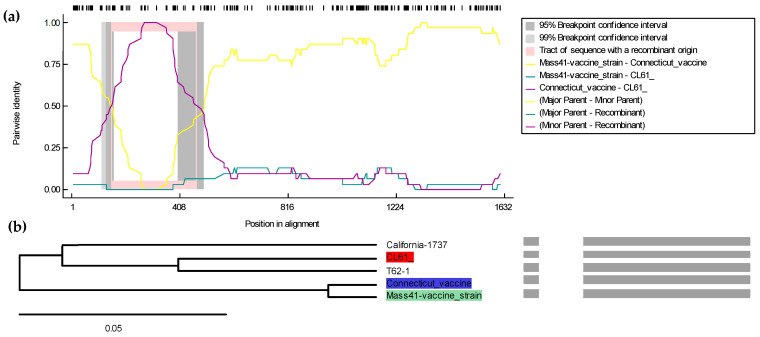
Recombinant analysis of the CL-61 isolate of the IBV based on the *S1 gene* sequence using the RDP5 software. In (**a**), the CL-61 strain of the IBV serves as the query sequence, while the lines show different pairs of analysis for recombination: yellow (Mass vaccine, Conn vaccine), green (Mass vaccine, CL-61), and purple (Conn vaccine, CL-61). In (**b**), the recombinant tree is presented, with the red area representing CL-61 IBV as the recombinant, the green area indicating the Mass41 vaccine-like (unknown) strain as the major parent, and the blue area showing T-62 as the minor parent of CL-61. Grey lines indicate regions of recombination. Dark grey areas represent consensus sequences. White areas highlight breakpoints, indicating specific points where recombination has occurred. The Shimodaira–Hasegawa tree topology test yielded a *p*-value of <0.0001, based on 100,000 bootstrap replicates.

Statistical algorithms were used to create a consensus tree based on the *S1 gene* nucleotide sequences. Interestingly, the analysis revealed that an unknown Mass-like IBV strain was the primary parent of the CL-61 strain of the IBV in the *S1 gene*, while the Conn vaccine strain, with a 100% nucleotide similarity, was determined to be the minor parent in the same gene. The multiple comparison probabilities, both corrected and uncorrected, were 1.157 × 10^−23^ and 1.157 ×10^−22^, respectively. The analysis based on the *S1 gene* showed nucleotide recombination at the starting and ending breakpoints in the *S1 gene* at nucleotide positions 150 and 473, respectively, with a 99% CI. The nucleotide region recombinant breakpoints contained two out of three HVRs of the *S1 gene* (Figure 5).

### 3.6. Pairwise Comparison of the S1 Glycoprotein Amino Acid Sequences

The aa sequences of the S1 glycoprotein for the IBV strains identified in this study were aligned and compared with the currently available vaccines in Canada. The identified viruses in this study shared different level of aa identity with the Conn and Mass (H120 strain) vaccines. The S1 glycoprotein of the CL-61 and T-62 IBV strains had 85.7% and 73.2% aa identities compared to the Conn vaccine, respectively. Compared to the S1 glycoprotein of the Mass vaccine, the CL-61 and T-62 strains shared 81.4% and 74% aa similarities, respectively. 

**Figure 6:**
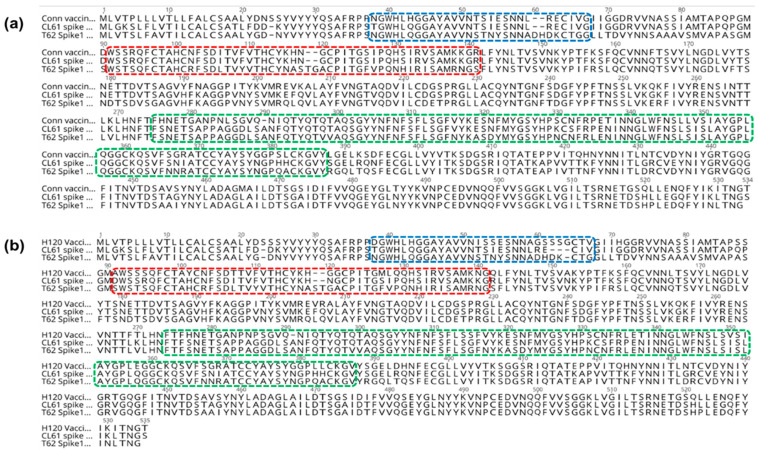
Pairwise alignment of the amino acid sequence of the S1 glycoprotein of the current IBV strains compared to the Conn (**a**) and Mass (**b**) vaccines. Amino acid changes in HVR 1 (amino acids 38–67) are indicated by the blue color, while the red and green colors refer to changes in the residues of HVR 2 (amino acids 91–141) and HVR 3 (amino acids 274–387), respectively.

There were 67 and 129 aa substitutions among the S1 glycoprotein of the CL-61 and T-62 IBV strains compared to the Conn vaccine, respectively. Importantly, two and nineteen of these aa variants were in HVR 1 and HVR 3 of the S1 glycoprotein of the CL-61 strain of the IBV, respectively. There were 10, 18, and 24 aa substitutions observed in HVR 1, HVR 2, and HVR 3 regarding the S1 glycoprotein of the T-62 strain of the IBV (Figure 6a).

Compared to the Mass vaccine, the S1 glycoprotein of the CL-61 and T-62 strains of the IBV exhibited 86 and 125 aa substitutions, respectively. Among these aa changes, there were ten, eight, and twenty-one aa substitutions in HVR 1, HVR 2, and HVR 3 of the S1 glycoprotein of the CL-61 strain of the IBV, respectively, while the S1 glycoprotein of the T-62 strain of the IBV demonstrated 12, 14, and 25 residue substitutions in HVR 1, HVR 2, and HVR 3, respectively (Figure 6b).

**Figure 7:**
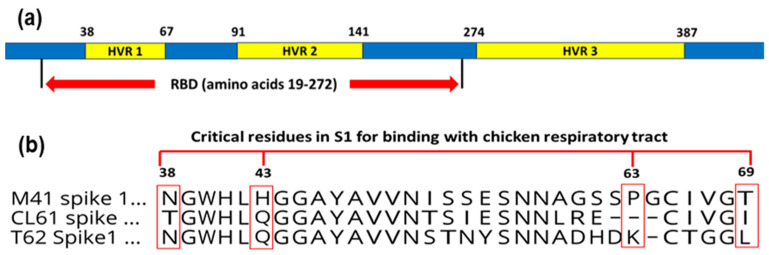
Identification of amino acid changes in the receptor-binding domain of the S1 glycoprotein (**a**) that are essential for virus–respiratory tract binding. (**b**) Alignment of the S1 glycoprotein revealed residue mutations in the current IBV isolates compared to the M41 IBV strain at positions 38, 43, 63, and 69.

The receptor-binding domain (RBD; Figure 7a) of the S1 glycoprotein involves aa (19–272) from HVR 1 and HVR 2. Furthermore, it has four residues, such as N38, H43, P63, and T69, which are vital for the binding of the S1 protein to the respiratory tract of chicken [52]. These aa are conserved among the S1 protein of the M41 strain of the IBV (Figure 7b). By comparing the S1 glycoprotein of current strains with the M41 IBV strain, N38 was conserved in the T-62 strain of the IBV. However, N38T substitution was evident in the CL-61 strain of the IBV. Residue substitutions were observed at positions 43 and 69 in both isolates. At position 63, the T-62 strain of the IBV had aa substitution (P63K), while the CL-61 strain of the IBV was identified by the existence of residue deletion.

### 3.7. Detection of the Amino Acids of the S1 Glycoprotein Under Selection Pressure

**Figure 8:**
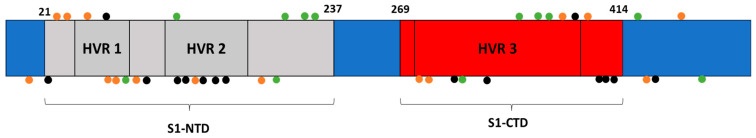
Schematic drawing of the entire S1 glycoprotein referring to the distribution of amino acids under negative selection (top) and episodic diversifying selection (bottom) in both IBV isolates. Top: orange circles show conserved amino acids under negative selection; green and black circles indicate unique residues under negative selection in the CL-61 and T-62 strains of the IBV, respectively. Bottom: orange circles depict conserved residues under episodic diversifying selection; green and black circles refer to distinct sites under episodic diversifying selection in the CL-61 and T-62 IBV isolates, respectively. S1-NTD: N-terminal domain of S1 glycoprotein; and S1-CTD: C-terminal domain of S1 glycoprotein. Homology modeling and verification of 3D structures of S1 glycoprotein.

The dN/dS ratio was calculated using FEL and FUBAR to assess whether residues of the S1 protein were subjected to pervasive diversifying (positive) or purifying (negative) selection pressures. No similar sites under positive selection pressure was detected by the utilized tools in the S1 protein of both IBV isolates. However, significant aa residues were identified under negative selection pressure in the S1 protein of the current IBV isolates. In the two IBV isolates, there were conserved aa residues at positions 27, 34, 46, 381, 396, and 507. Nevertheless, unique aa residues at positions 113, 200, 225, 231, 331, 366, 373, and 459 were found in the S1 protein of the CL-61 strain of the IBV, while two residues at positions 54 and 386 were observed in the T-62 strain of the IBV. Overall, these sites were distributed mainly in and/or proximate to the three HVRs and C-terminal end (CTD) in the CL-61 strain of the IBV, while they were localized predominantly in and/or near the HVR 1 and HVR 3, as well as the CTD (Figure 8).

Furthermore, episodic diversifying selection was analysed using the MEME method. There were 13 and 22 significant sites in the S1 glycoprotein of the CL-61 and T-62 strains of the IBV, respectively. Among these sites, nine aa residues were conserved in both IBV isolates and they were located at positions 14, 54, 64, 71, 127, 172, 276, 283, and 434. Sites at 66, 193, 301, and 469 were exclusive to the CL-61 IBV isolate, whereas positions at 24, 88, 117, 120, 129, 142, 157, 300, 346, 400, 404, 405, and 436 were unique to the T-62 IBV isolate. These sites were located mainly in both domains of the S1 glycoprotein (NTD and CTD), including the HVRs (Figure 8).

### 3.8. Homology Modeling and Verification of 3D Structures of the S1 Glycoprotein

**Figure 9:**
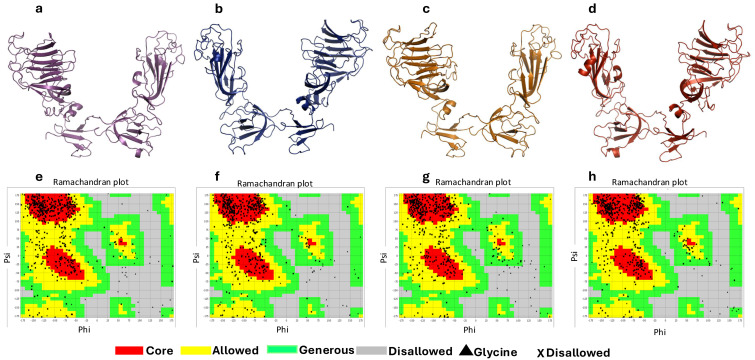
Three-dimensional structures (**a**–**d**) and Ramachandran plots (**e**–**h**) of S1 glycoprotein. CL-61 (**a**,**e**); T-62 (**b**,**f**); DMV/1639 (**c**,**g**); and Mass/SES (**d**,**h**). The 3D constructs were built by the I-TASSER server and viewed in the PyMOL software, while the Ramachandran plots were generated by the Vadar server. Prediction of potential N-glycosylation sites in S1 glycoprotein.

The 3D structures of the S1 glycoprotein of the current isolates and two Canadian IBV isolates (DMV/1639 and Mass/SES) were generated using the I-TASSER server (Figure 9a–d). This server predicted five models; each model’s confidence level was quantified by the C-score, which was derived from the impact of the threading templates alignment and convergence parameters in simulations. In general, the C-score ranged from −5 to 2, where higher values indicate a more confident model. Furthermore, the TM-score is another parameter, which is primarily based on the C-score and protein length. The models with the highest C-scores and TM-scores were selected to ensure a better quality of the used 3D structures (Supplementary Table S5). In terms of the similarity between the current isolates and currently circulating Canadian IBV strains, the 3D structure of the S1 glycoprotein of the CL-61 strain of the IBV shared 57.16% and 30.25%, compared to that of the DMV/1639 and Mass/SES strains of the IBV, respectively. On the other hand, the 3D model of the S1 glycoprotein of the T-62 strain of the IBV showed a lower identity, representing 16.14% and 12.41% compared to the DMV/1639 and Mass/SES strains of the IBV, respectively.

In addition, the quality of the refined 3D structures was evaluated using a Ramachandran plot generated by the Vadar server. The obtained Ramachandran plot demonstrated that the majority of aa residues of the current constructs localized in the most favored (core) and allowed regions, representing 91.7%, 93.4%, 91.2%, and 93.2% in the case of the S1 glycoproteins of the CL-61, T-62, DMV/1639, and Mass/SES strains of the IBV, respectively (Figure 9e–h). 

### 3.9. S1 Glycoprotein N-Glycosylation Sites Prediction

**Figure 10:**
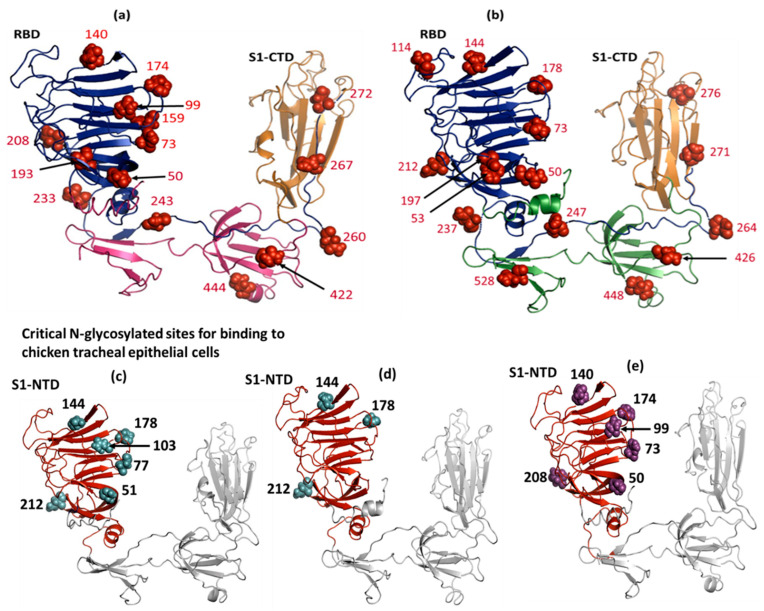
Three-dimensional structure of the complete S1 glycoprotein, highlighting the N-glycosylation in CL-61 (**a**) and T-62 strains of IBV (**b**). The N-glycosylated amino acids (indicated by red spheres) were predicted by the NetNglyc server. These sites were distributed in different portions of the S1 glycoprotein including the RBD (shown by the blue color) and S1-CTD (highlighted by the golden yellow). Six N-glycosylation sites at S1-NTD (indicated by the red color) are essential for the binding to epithelial cells of trachea (**c**–**e**). All these critical sites were maintained in the S1-NTD of the Mass/SES strain of the IBV ((**c**); indicated by cyans), while three out of six were found in the IBV T-62 S1-NTD ((**d**); shown by cyan). The S1-NTD of the CL-61 IBV strain had some N-glycosylated residues close to the previous sites ((**e**); shown by magenta). The PDB files of the S1 glycoproteins were viewed in the PyMOL software. Molecular docking of S1 glycoprotein against sialic acid.

The prediction of N-glycosylation sites was performed with the consensus sequence (sequon) Asn-X-Ser/Thr, in which Asn corresponds to asparagine, X corresponds to any aa except proline (Pro), Ser corresponds to serine, and Thr corresponds to threonine. A total of 15 and 16 N-glycosylated sequons (Asn-X-Ser/Thr) were identified among the IBV CL-61 and T-62 S1 glycoproteins, respectively. Most of these sites were in the RBD including HVR 1 and HVR 2, as well as being situated in and/or near to S1-CTD, including HVR 3 (Figure 10a,b). It has been shown that an interaction with the tracheal epithelial cells of chickens is dependent on N-glycosylation at positions 51, 77, 103, 144, 178, and 212 in S1-NTD [8]. These N-glycosylation sites were conserved in the S1-NTD of the S1 glycoprotein of Mass/SES strain of the IBV (Figure 10c), which was used as a comparison to the S1 glycoprotein of the current IBV isolates. Among the IBV T-62 S1-NTD glycoproteins, the exact positions at 144, 178, and 212 existed, while the other sites (51, 77, and 103) were absent (Figure 10d). Instead, the S1-NTD glycoprotein of IBV CL-61 lacked these sites; however, it was characterized by the occurrence of N-glycosylated sites at positions 50, 73, 99, 174, 140, and 208, which were in proximity to the sites (Figure 10e).

### 3.10. Molecular Docking of the S1 Glycoprotein Against Sialic Acid

Sialic acid receptors on the epithelial surface of the host tissue have been involved in initiating IBV infection, particularly at an early stage [53,54,55]. Therefore, molecular docking was used to determine the binding affinity of the S1 protein against sialic acid residues. In comparison with the existing Canadian IBV strains, DMV/1639 and Mass/SES, the potency of the S1 glycoprotein in binding sialic acid was evaluated in the current IBV isolates. The findings showed that the S1 glycoproteins of the CL-61 and T-62 strains of the IBV demonstrated binding energies of −6.8 kcal/mol and −6.7 kcal/mol, respectively, while the S1 glycoproteins of DMV/1639 and Mass/SES had binding energies of −6.2 kcal/mol and −6.4 kcal/mol against sialic acid, respectively. Hence, the two current IBV isolates possessed a higher affinity for their ligand (sialic acid) than these Canadian IBV strains. 

The molecular interactions between the IBV CL-61 S1 glycoprotein and sialic acid exhibited five conventional and two carbon hydrogen bonds with ASP 22, ASP 23, and ASN 193. Nevertheless, non-bond contacts were also evident, including van der Waals bonds at residues THR 19, LEU 20, VAL 48, THR 51, ILE 53, THR 80, ALA 81, PRO 82, GLN 83, PRO 191, VAL 192, and SER 195 (Supplementary Figure S1a). In terms of the interactions of the IBV T-62 S1 glycoprotein with sialic acid, there were four conventional hydrogen bonds with LYS 151, ARG 156, and ASP 172. However, the non-bond connections of van der Waals were observed at positions TYR 29, ARG 34, TYR 152, ILE 154, SER 157, LEU 154, SER 157, LEU 169, ASN 170, GLYC 171, THR 214, and VAL 215 (Supplementary Figure S1b). The binding of the S1 glycoprotein of DMV/1639 with sialic acid revealed six hydrogen bonds at residues THR 10, ALA 14, GLN 167, SER 169, and TYR 171. On the other hand, the non-bond bindings of van der Waals could be detected at positions LEU 11, PHE 13, LEU 15, TYR 29, SER 32, ASN 167, and LEU 176 of the DMV/1639 S1 glycoprotein (Supplementary Figure S1c). While the S1 glycoprotein of Mass/SES bounded to sialic acid with five hydrogen bonds at amino acids SER 262, THR 265, ARG 420, and ASN 422, van der Waals non-bond interactions were seen at residues GLU 260, ASN 261, VAL 263, ASN 264, THR 267, HIS 420, TYR 423, GLN 478, and GLU 479 of the Mass S1 glycoprotein (Supplementary Figure S1d). 

## 4. Discussion

It was noted as early as 1956 and experimentally demonstrated that there is a lack of cross-protection among different IBV strains [56], although coronaviruses including the IBV have an exoribonuclease domain known as Nsp 14. The Nsp 14 is a part of the replicase complex and involved in the proofreading mechanism; the average rate of synonymous mutation is around 1.2 × 10^−3^ substitutions per site per year [57]. The IBV has the potential to change either through spontaneous mutation, where merely a few aa changes in the S protein can generate new variants, or through genetic recombination [6]. Subsequently, new genotypes, serotypes, and protectotypes emerge that can evade host immunity and vaccination efforts, further complicating IB control measures [10,11].

The molecular analysis of the IBV variants from infected Canadian poultry revealed that these variants are unique with significant variations in both nucleotide and aa sequences compared to the known IBV strains. First, we discovered that these two IBV variants could be classified under genotype 1 between lineage 25 and lineage 7. Second, we observed that both these IBV variants have undergone recombination involving wild-type and vaccine IBV strains. Third, these variants are under constant negative pressure leading to the purification of the variants in the host. Fourth, these unique variants have a higher number of N-glycosylation sites and higher sialic acid binding affinity in their S1 HVRs compared to the currently circulating Canadian DMV/1639 and Mass (SES) IBV strains. This study is significant in understanding the genetic dynamics of the IBV, including adaptation and purification. Unique variants can potentially result in the emergence of well-established IBV strains, such as the IBV DMV/1639 strain, which is a continuous threat to the poultry industry in Eastern Canada.

In this study, the minimum variation in the complete *S1 gene* nucleotide sequences of the T-62 and CL-61 strains of the IBV was 14.24% and 13.08%, respectively, compared to the 114 sequences of various IBV strains including vaccine strains, strains common in Canada and the US, and representative strains from all lineages and genotypes. According to the new (2016) criteria of classification, both variants are unique [14]. Similarly, differences in the aa sequences of the T-62 and CL-61 strains of the IBV were 15.7% and 14.05%, respectively. Both unique variants from our study have a common ancestor but are located on different branches showing variation between themselves. In agreement with our observation, a similar phenomenon has previously been observed in several geographical locations. For example, five unique IBV variants have been isolated and matched with the aa sequences of the prevalent Conn, Arkansas 99, and California strains [58]. As much as 23.4% variation was noted in the S1 aa, indicating the continuous emergence of unique variants in the USA [58]. Similarly, in the UK, unique QX-type IBV isolates were isolated from outbreaks in commercial broiler flocks [59]. Several sub-types of the Arkansas IBV with antigenic diversity were isolated from the broiler flocks in Delmarva [60]. Unique IBV variants were isolated with higher evolutionary distances between the isolates and the commercial vaccines (GI-1). The complete genome sequence similarity of the Canadian IBV DMV1639 strain was around 94% with the Conn vaccine strain [2]. 

IBV recombination can take place among various genotypes and lineages. Both wild-type and vaccine IBV strains can be involved in the recombination process [40]. Recombination is more common in single-strand RNA viruses such as the IBV and other coronaviruses [61]. There is abundant IBV literature with reports from various parts of the world documenting recombination events in the IBV genome. Most of these recombination events have occurred between a vaccine strain and wild-type IBV. In the USA, eight IBV isolates were analyzed, and evidence of recombination was found in the entire genome [62]. Another study revealed that the IBV Ark strain has acquired epitopes from the Mass strain through mutation [63]. A retrospective analysis showed that the N1/88 IBV isolate from Australia received some part of its *S1 gene* from an unknown avian coronavirus in the 1980s [9]. More than 215 recombination events were reported from Europe, including some IBV strains with multiple recombination breakpoints in their genome [40]. Adaptation of the D181 strain was observed when it converted from a low-level incidental strain in 2017 to the second highest prevalent strain in 2018 in Dutch layer and breeder flocks [13]. Similarly, recombination events in different IBV strains, such as KM91, QX, K40/09, and IBV/Korea/48/2020 from South Korea and CK/CH/MY/2020, ck/CH/LGX/130530, tl/CH/LDT3-03, 202109, CK/CH/SCMY/160315, YX10, and LJL/08-1 from China, have been documented [33,64,65,66,67,68,69,70]. Unique recombinant IBV variants have been isolated from backyard poultry in Kenya [7]. Similarly, recombinant IBV strains have emerged and are prevalent in China [8]. From India, a 4/91-like unique nephropathogenic IBV variant was isolated from an IBV outbreak in chickens [71]. Two recombinant IBV isolates were documented in a case report from Saudi Arabia [41]. Finally, an investigation of Japanese field IBV isolates targeting the hypervariable region in the S1 subunit has led to the identification of a previously unreported IBV variant [72].

Both structural and Nsps are potential sites for recombination. Most of these variations are found in the *S gene*, reported in 54% of the reviewed studies (15 out of 28 references) followed by 1ab polyprotein 35.7%, (10 out of 28 references) [73,74]. Recombination breakpoints have been found in the *nsp2*, *nsp3*, *nsp16*, and *S genes* [9]. In another analysis, recombination was detected in the *S*, *E*, *M*, and *N genes* of 4/91 IBV strain [67]. Multiple recombination in different parts of the *1ab gene*, encoding for the *nsp2*, *nsp3*, *nsp8*, and *nsp12 gene* regions, were detected [33,40]. Two independent recombination events were noted in the *S2* and N genes of the CU-T2 IBV isolate [75]. 

Recombination can lead to the rise of viruses with a changed affinity for tissues and pathogenicity. When tested in SPF chickens, IBV isolate LJL/110302 has a broader tissue tropism and a more rapid replication rate compared to LLN/111169 [76]. Similarly, with the use of the routine vaccine, the birds were not protected from the infection of the recombinant IBV strain. The recombinant strain is more virulent [9,67]. A mortality of 30–40% was observed in day-old chicks experimentally infected with a recombinant IBV isolate [33]. Finally, another study found that recombinant isolate I0305/19 is a highly nephropathogenic strain [69]. Genomic analysis revealed the recombination of the Canadian IBV-DMV/1639 with the Conn and 4/91 vaccine strains and another unidentified strain [2]. The Canadian DMV/1639 is highly pathogenic to chickens, particularly laying birds. The isolate leads to around 46% cystic lesions in the oviduct observed in the 16th week, later turning into false layer syndrome [77]. DMV/1639 dropped egg production in 29-week layers by 40% on the fifth-day post-infection (dpi) [78].

The T-62 strain of the IBV is a potential recombinant of the Iowa state and Conn vaccine strains. A recombinant fragment was noted in the *1ab gene*. Interestingly, the *S1 gene*-based analysis revealed that a fragment has also recombined with the Georgia strain (GA/10216). The CL-61 strain of the IBV is a potential recombinant strain with the major parent of its genome coming from the Conn vaccine strain and the minor parent from the IBV T-62 isolate of this study. An unknown Mass-like strain was the primary parent of the CL-61 strain of the IBV in the *S1 gene*.

Vaccinal strains such as Mass and Conn were associated in recombination events in both unique variants of this study. This may be because live vaccines containing the Mass and Conn strains are the only licensed vaccines against IBV infection in Canada [14,79] and are used widely for the control of IB in poultry flocks. In addition, recombination with other infectious stains from the Iowa and Georgian states of the USA was found. The Iowa strain (IBV-IA1162/2020) was isolated from a layer chick outbreak in Iowa, whereas [80] the Georgian (IBV-GA/10216/2010) strain was found in clinically affected broilers and backyard flocks in Georgia [81]. Wild bird movements might have a role in spreading IBV strains that are closely related to the USA origin [82]. US-like variants of IBV were a major portion of the diagnostic samples from the 2000s processed at the Animal Health Laboratory, University of Guelph, Ontario, Canada [35], suggesting IBV transmission across the border is possible. Finally, Canada imports day-old chicks and poultry products from the US, which can be another potential source of IBV [83]. 

A range of studies have focused on the analysis of the N-glycosylation sites in different IBV strains. Various strains either lose or gain N-glycosylation sites in the S1 protein when compared to the other IBV strains [84,85]. Major variations were observed in the N-glycosylation and phosphorylation motifs among the DMV/1639 IBV [86]. In the current analysis, most of the N-glycosylation sites were in the RBD, including HVR 1 and HVR 2. The N-glycosylation at positions 51, 77, 103, 144, 178, and 212 is important in binding to the chicken tracheal epithelium [8]. In the T-62 isolate of the current study, N-glycosylation was detected at positions 144, 178, and 212, while the other sites (51, 77, and 103) were absent. On the other hand, the S1 glycoprotein of the IBV CL-61 had substitution sites with an equal number of motifs (50, 73, 99, 174, 140, and 208). Likewise, four (N212, N237, N247, and N276) N-linked glycosylation sites in the S protein of the IBV were confirmed in the proteomic study. N to D/Q mutations in N212 and N276 were found to eliminate the infectivity of the recombinant IBV [87]. The N-glycosylation motifs in the West Nile virus play a role only in regulating the virus assembly and release in the replication and have little effect on the infectivity of the virus [88]. In contrast, HIV infections showed that N-glycans play an important role in the infectivity of the virus. N-glycosylation-missing proteins showed a loss of viral infectivity [89]. The mutation in the N-linked glycosylation site in Salmonid Alphavirus (SAV) produced an attenuated strain of the virus with no virulence [90]. The envelope proteins of the envelope viruses, such as HIV, influenza virus, IBV, SARS, and SARS-CoV, are deeply glycosylated. The glycans can mask the antigenic epitopes of the virus that can ultimately avoid recognition by neutralizing antibodies and lead to immune escape [32]. Overall, it appears that the CL-61 isolate, with its N-glycosylation variation, might have a different affinity for host receptors. The variation and abundance of sialic acid compared to the M41 and T-62 strains could potentially make CL-61 more pathogenic. However, in vivo animal experiments are required to validate these findings. 

Mutation in the sialic acid binding domain from one IBV strain into another can alter the virulence, antigenic property, and tissue tropism indicating strain-specific variations [91]. The elimination of sialic acids through treatment of *Arthrobacter ureafaciens* neuraminidase (AUNA) eliminated the binding of the M41 IBV strain. These results suggest that the glycosylation of the RBD plays a role in its binding to the sialic acid receptors on chicken cells [92]. The two current IBV isolates possessed a higher affinity for their ligand (sialic acid) than the Canadian DMV/1639 and Mass/SES IBV strains. In contrast, according to [86], the sialic acid binding affinity of the mutant IBV isolates was significantly lower than the wild-type isolate. Although the analysis indicates that both isolates of this study have a higher sialic acid binding affinity compared to the well-established pathogenic IBV strains in Canada (DMV/1639, Mass SES), many other factors such as the host response, immunity, vaccine efficacy, and replication rate may also influence virulence. 

Significant aa residues were identified to be under negative selection pressure in the S1 protein of the current IBV isolates. Two essential forces, genetic variation and selection, are associated with the apparent virus evolution [5]. Negative or purifying selection pressure reduces the variability in the population and positive selection pressure increases the rate of mutation by enhancing variability in the population [93]. Hypervariable regions in the S1 protein are more prone to positive selection pressure. Amino acid residues experiencing positive selection pressure were found in the Italy 02 IBV strain [94]. Selective forces within the S1 protein of the QX strain were enhanced due to the selective pressure exerted by using the homologous vaccines [95]. In silico analysis revealed constant selection pressure on the unique IBV variants [41,96]. Positive selection pressure can be attributed to factors other than host resistance, such as biosecurity measures and the use of disinfectants [5,74]. In contrast to positive selection pressure, negative selection pressure or purifying selection promotes fewer optimally adapted strains of the virus and constrains genetic diversity [97,98]. Furthermore, the use of unsuitable vaccines, the presence of immunosuppressive agents, and partial immunization can contribute to increased virus circulation, creating selection pressure and the generation of variants capable of evading the host immune response [99]. 

This study was limited to samples from only two outbreak sites. Access to samples from additional outbreaks would have strengthened the findings. To further investigate the pathogenicity of these unique isolates, future research should focus on isolating the virus in embryonated eggs and conducting controlled in vivo studies using various types of specific pathogen-free (SPF) chickens.

## 5. Conclusions

In conclusion, both spontaneous mutation and genetic recombination play crucial roles in the evolution of the virus. The genome of the IBV has the potential for continuous emergence of new IBV variants. Unique IBV variants with significant evolutionary distances from commercial vaccines have been identified impacting Canadian poultry flocks. Cross-border transmission between Canada and the USA, the role of selection pressure, biosecurity practices, and vaccination strategies drive IBV evolution.

## Data Availability

The sequencing data of the manuscript are submitted and available at NCBI with accession numbers. All the primary data analysis in the production of this manuscript will be available on request and are saved by the first author of the manuscript.

## Supplementary Materials

The following supporting information can be downloaded at https://www.mdpi.com/article/10.3390/genes15111480/s1; Table S1: Table of reference IBV sequences representing all genotypes and lineages used as reference for alignment; Table S2: Genomic features of the unique IBV isolates (CL61 and T62); Table S3: Confirmation table from RDP5 analysis with significant p-values and event status based on both whole genome and S1 gene sequences; Table S4: Confirmation table from RDP5 analysis with significant p-values and event status based on both whole genome and S1 gene sequences; Table S5: C-score and TM-score of the obtained 3D structures of S1 glycoprotein of current and two Canadian IBV isolates (CL61 and T62). Figure S1: S1 glycoprotein-sialic acid interactions were analyzed in CL61 (a), T62 (b), DMV/1639 (c), and Mass/SES (d). The 3D structures of S1 glycoproteins were viewed in the PyMOL software, while 2D structures with highlighting the bonds between sialic acid and amino acids of S1 glycoprotein were created by the Discovery Studio. Ligand (sialic acid) is shown by cyan spheres. For bonds, conventional and carbon hydrogen bonds were indicated by intense green and light green, respectively, while van der Waals demonstrated no connections. Each amino acid included in the interactions was identified with three letters and a number.
