# Supplementary material for: Nucleotide and Amino Acid Analyses of Unique Infectious Bronchitis Virus (IBV) Variants from Canadian Poultry Flocks with Drop in Egg Production
Source: Genes (Basel). 2024 Nov 17;15(11):1480. doi: 10.3390/genes15111480 (PMC11593648; doi:10.3390/genes15111480)
Supplement: Supplementary file 1 [file genes-15-01480-s001.zip › genes-3313420-supplementary.pdf]

Supplementary table S1: Table of reference IBV sequences representing all genotypes and lineages used as reference for alignment.

IBV Sequence	Accession Number	Country
ArkDPI	AF006624	USA
CV-56b	AF027509	USA
PA/Wolgemuth/98	AF305595	USA
CU82792	AF317214	USA
GA/13055/00	AF338719	USA
PA/171/99	AF419314	USA
M41	AY561711	USA
CU510	AY561716	USA
PA/5344/98	AY789947	USA
CAL99	DQ912831	USA
Conn46 1996	FJ904716	USA
H120	FJ888351	USA
DMV/5642/06	EU694402	USA
CA/1737/04	EU925393	USA
Arkansas Vaccine	GQ504721	USA
GA08	GU301925	USA
Iowa 97	GU393337	USA
L905	JQ964070	USA
GA/13384/2013	KM660635	USA
GA/10216/2010	KM660636	USA
GA/13485/2013	KP085597	USA
MDL_DMV1639	KX529720	USA
Gray	L14069	USA
JMK	L14070	USA
Holte	L18988	USA
Beaudette	M95169	USA
ARK99	M99482	USA
SE17	M99484	USA
FLS/AZ/17	OM525798	USA
DE/072/92	U77298	USA
Connecticut vaccine	KF696629	USA
Holte	L18988	USA
IBV/Ck/Can/18-T-62	***PP737794***	***Canada***
IBV/Ck/Can/18-CL-61	***PP783617***	***Canada***
IBV/Ck/Can/18-049707	MN512438	Canada
IBV/Ck/Can/17-036989	MN512435	Canada
BV_SES_15AB-01	MH539771	Canada
IBV/Ck/Can/17-036989	MN512435	Canada

IBV/Ck/Can/18-049707	MN512438	Canada
Qu_mv	AF349621	Canada
Qu16	AF349620	Canada
CK/CH/LLN/111169	KF411040	China
gammaCoV/ck/China/I0111/14	KY407557	China
R22-452490	MW896952	China
CK/CH/JX/JA09-1	HQ18890	China
SDIB781/2012	KF007209	China
ck/CH/LGX/111119	KX640829	China
gammaCoV/ck/China/I0636/16	MH924835	China
CK/CH/SC/MS11	KJ524606	China
GX-YL130025	KJ999795	China
GX-NN130021	KM365468	China
CK CH GX NN1111 S1	KC692317	China
CK/CH/LJL/111054	JQ739329	China
CK/CH/LHLJ/110664	JQ739299	China
ck/CH/LSD/110857	JQ739375	China
ck/CH/LSD/110712	JQ739363	China
48SD-96VI	KC577388	China
QS	JQ250818	China
gammaCoV/ck/China/I0111/14	KY407557	China
gammaCoV/ck/China/I0118/14	KY407558	China
QXIBV	AF193423	China
Q1	AF286302	China
GX2-98	AY251816	China
SDW	DQ070840	China
SAIBK	DQ288927	China
TC07-2-	GQ265948	China
variant 1	AF093795	Israel
variant 2	AF093796	Israel
IS/1201	DQ400359	Israel
RF/01/02	AJ441314	Russia
FR-85131-85	AJ618985	France
JP8443	AY296745	Japan
TP/64	AY606320	Taiwan
Feb_94	AY606324	Taiwan
3381_06	GQ229245	Taiwan
Spain/98/313	DQ064808	Spain
gCoV/Ck/ES/AC-11764/01/2021	OM746676	Spain
Spain/00/336	DQ386098	Spain
V18/91	U29521	Australia
V2-02	DQ490215	Australia
N1/62	U29522	Australia

N4/02	DQ59618	Australia
N1/08	JN176213	Australia
N5/03	DQ59619	Australia
IZO 28/86	KJ941019	Italy
It/497/02	DQ901377	Italy
Moroccan-G/83	EU914938	Morocco
NGA/N544/2006	FN182269	Nigeria
NER/28/2007	FN182272	Nigeria
NGA/295/2006	FN182276	Nigeria
NGA/324/2006	FN182277	Nigeria
SNU8067	JQ977697	South Korea
K620/02	FJ807944	South Korea
18RS1461-1	MN696789	Trinidad and Tobago
18RS1461-5	MN696792	Trinidad and Tobago
18RS1461-7	MN696793	Trinidad and Tobago
gCoV/Ck/DE/AC-14235/06/201	OM746672	Germany
gammaCoV/ck/DE/AC-14235/06/201	OM746672	Germany
CK/DE/IB80/2016	MT591566	Germany
B1648	X87238	Belgium
18RS1461-8	MN696794	Maxico
BL-56	AF352831	Mexico
IBV/Brasil/351/1984	GU393339	Brazil
UFMG/1141	JX182783	Brazil
IBV422	KF809791	India
V25	KF757451	India
H/IBV/2016/CU-96	MG191016	Thailand
6/82	X04723	UK
Eg/1265B/2012	KC533682	UK
UK/7/91	Z83975	UK
UK/L-633/04	DQ901376	UK
4/91 vaccine	KF377577	UK
CK/CH/LDL/97I	EF030995	The Netherlands
D1466	M21971	The Netherlands
D274	X15832	The Netherlands
V1397	M21968	The Netherlands

Supplementary table S2: Genomic features of the unique IBV isolates (CL61 and T62).

Region of the Genome	CL-61 isolate			T-62 isolate		
	Starting nt - Ending nt	No. of nt	No. of aa	Starting nt - Ending nt	No. of nt	No. of aa
5′UTR	1 - 472	472		1 - 496	496	
1ab	472 - 20,364	19893	6630	496 - 20,390	19896	6631
S	20,315 - 23,794	3480	1159	20,341 - 23,832	3492	1163
3a	23,794 - 23,967	174	57	23,832 - 24,005	174	57
3b	23,967 - 24,161	195	64	24,005 - 24,199	195	64
E	24,142 - 24,483	342	113	24,180 - 24,488	309	102
M	24,442 - 25,113	672	223	24,457 - 25,137	681	226
4b	25,115 - 25,399	285	94	25,138 - 25,422	285	94
4c	25,320 - 25,490	171	56	25,343 - 25,513	171	56
5a	25,474 - 25,671	198	65	25,497 - 25,694	198	65
5b	25,668 - 25,916	249	82	25,691 - 25,939	249	82
N	25,859 - 27,088	1230	409	25,882 - 27,111	1230	409
6b	27,097 - 27,303	207	68	27,120 - 27,344	225	74
3′UTR	27,304 - 27,577	273		27,112 - 27,611	500	

Supplementary table S3: Confirmation table from RDP5 analysis with significant *p*-values and event status based on both whole genome and S1 gene sequences.

Methods	Based on the whole genome sequence		Based on the S1 gene sequence	
	Average *p*-value	Event status	Average *p*-value	Event status
RDP (R)	3.320×10^{-26}	+	7.128×10^{-12}	+
GENECONV (G)	9.133×10^{-20}	+	4.644×10^{-10}	+
BootScan (B)	2.426×10^{-17}	+	3.173×10^{-11}	+
MaxChi (M)	1.127×10^{-27}	+	1.480×10^{-12}	+
Chimeara (C)	6.902×10^{-21}	+	3.561×10^{-11}	+
SiScan (S)	3.513×10^{-28}	+	2.722×10^{-27}	+
3Seq (T)	1.713×10^{-06}	+	6.795×10^{-09}	

Supplementary table S4: Confirmation table from RDP5 analysis with significant *p*-values and event status based on both whole genome and S1 gene sequences.

Methods	Based on the whole genome sequence		Based on the S1 gene sequence	
	Average *p*-value	Event status	Average *p*-value	Event status
RDP (R)	8.670×10^{-16}	+	1.157×10^{-22}	+
GENECONV (G)	6.236×10^{-11}	+	5.871×10^{-21}	+
BootScan (B)	6.520×10^{-13}	+	5.768×10^{-12}	+
MaxChi (M)	2.888×10^{-11}	+	4.905×10^{-11}	+
Chimeara (C)	3.421×10^{-14}	+	2.321×10^{-10}	+
SiScan (S)	8.735×10^{-18}	+	1.625×10^{-11}	+
3Seq (T)	1.554×10^{-14}	+	5.330×10^{-21}	

Supplementary table S5: C-score and TM-score of the obtained 3D structures of S1 glycoprotein of current and two Canadian IBV isolates (CL61 and T62).

**S1 glycoprotein**	**C-score**	**TM-score**
CL61	0.20	0.70±0.12
T62	0.66	0.76±0.10
DMV/1639	0.17	0.71±0.12
Mass/SES	0.38	0.76±0.10

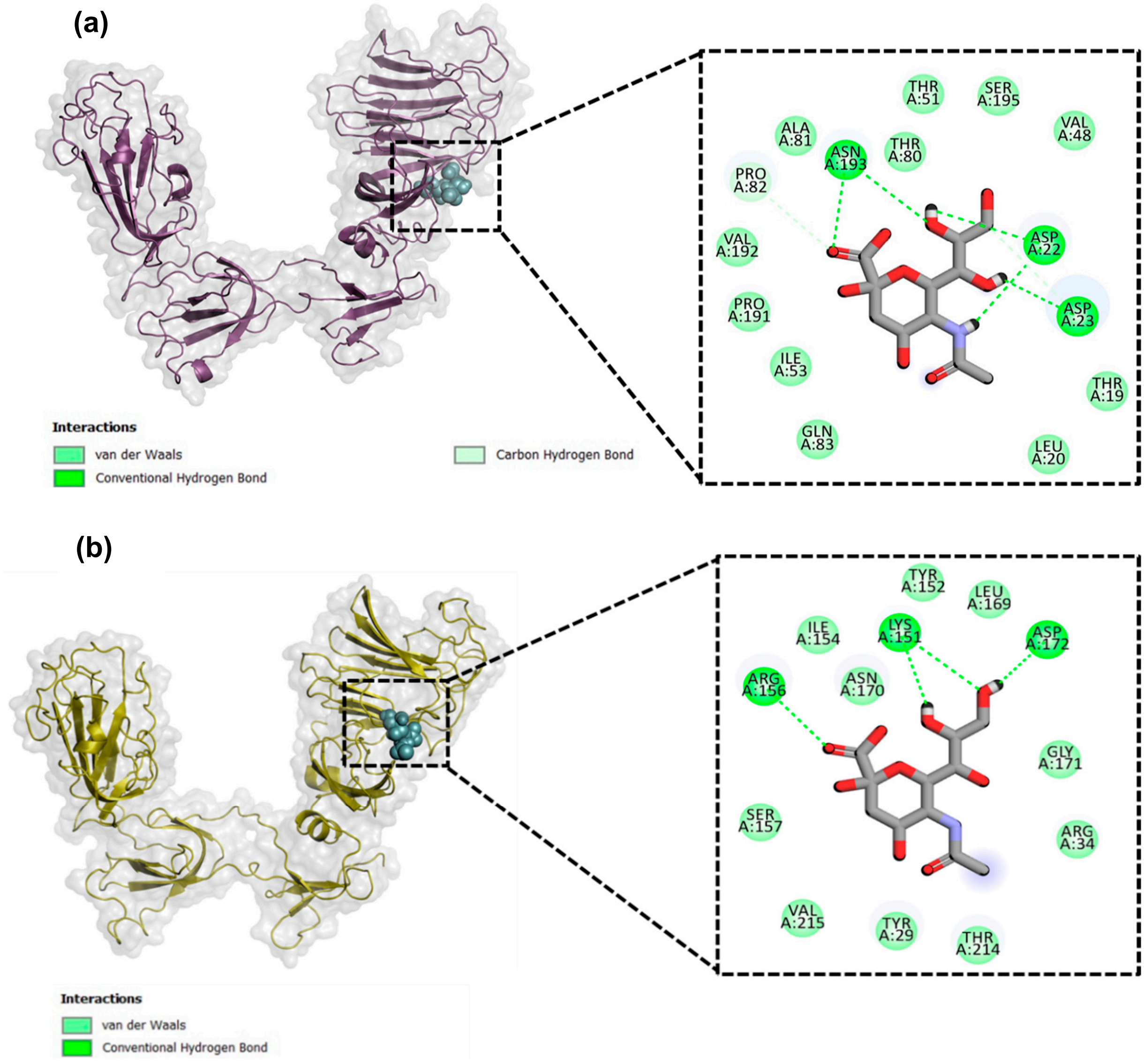
(a)
THR A:51
SER A:195
VAL A:48
ALA A:81
ASN A:193
THR A:80
PRO A:82
ASP A:22
VAL A:192
ASP A:23
PRO A:191
ILE A:53
THR A:19
GLN A:83
LEU A:20
Interactions
van der Waals
Conventional Hydrogen Bond
Carbon Hydrogen Bond
(b)
TYR A:152
LEU A:169
ILE A:154
LYS A:151
ASP A:172
ARG A:156
ASN A:170
GLY A:171
SER A:157
ARG A:34
VAL A:215
TYR A:29
THR A:214
Interactions
van der Waals
Conventional Hydrogen Bond

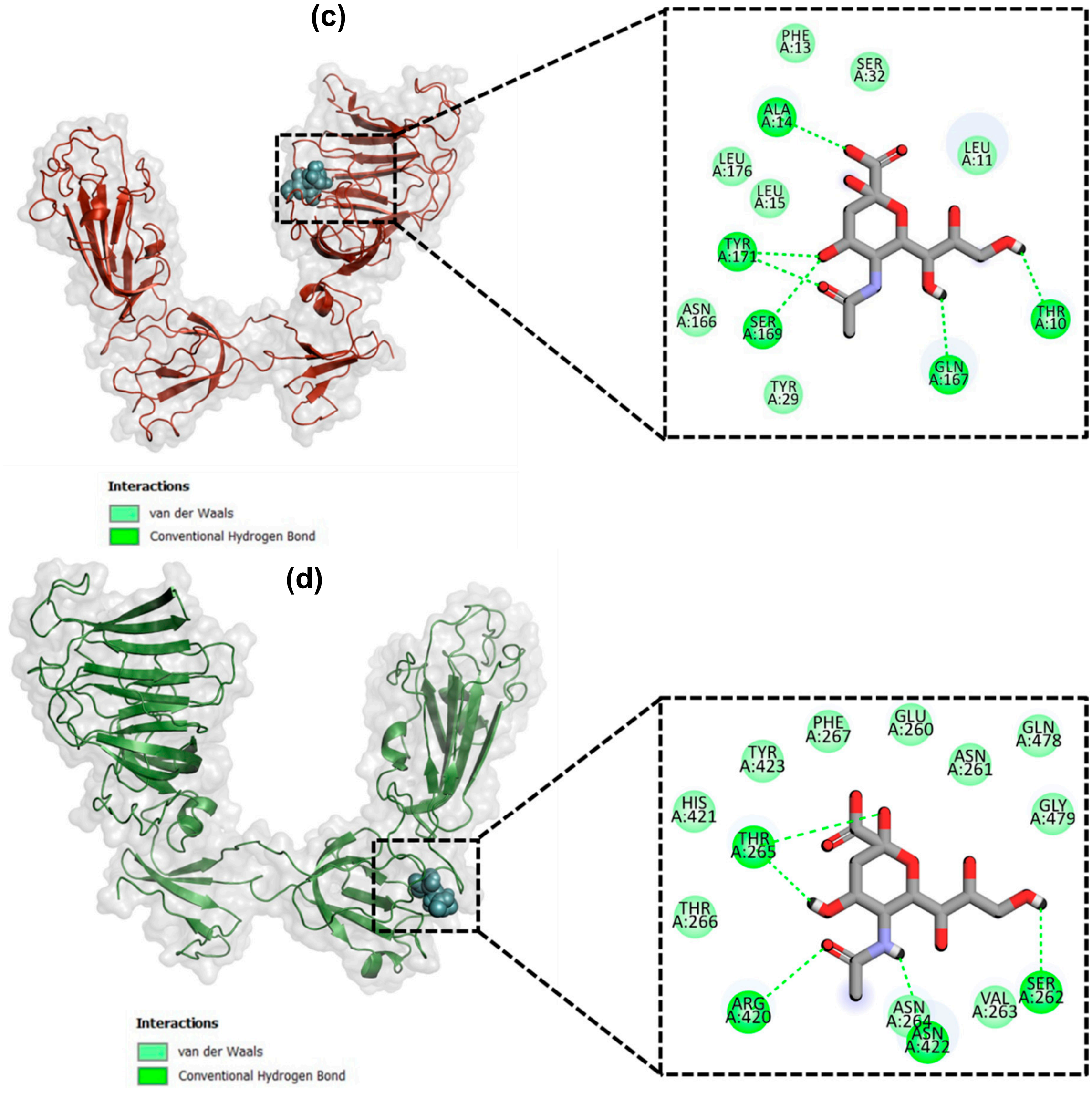

Supplementary figure S1. S1 glycoprotein-sialic acid interactions were analyzed in CL61 (a), T62 (b), DMV/1639 (c), and Mass/SES (d). The 3D structures of S1 glycoproteins were viewed in the PyMOL software, while 2D structures with highlighting the bonds between sialic acid and amino acids of S1 glycoprotein were created by the Discovery Studio. Ligand (sialic acid) is shown by cyan spheres. For bonds, conventional and carbon hydrogen bonds were indicated by intense green and light green, respectively, while van der Waals demonstrated no connections. Each amino acid included in the interactions was identified with three letters and a number.
